# Gabapentin CNS exposure and analgesic response are modulated by OCT2 genotype in patients with chronic neuropathic pain

**DOI:** 10.3389/fphar.2026.1760901

**Published:** 2026-03-12

**Authors:** Lina Zhou, Priscila A. Yamamoto, Melody Walker, Ana Carolina Conchon Costa, Gabriela R. Lauretti, Fabiola Dach, Stephan Schmidt, Natalia Valadares de Moraes

**Affiliations:** 1 Center for Pharmacometrics and Systems Pharmacology, College of Pharmacy, University of Florida, Orlando, FL, United States; 2 School of Medicine of Ribeirao Preto, University of Sao Paulo, Ribeirao Preto, Sao Paulo, Brazil

**Keywords:** gabapentin, neuropathic pain, OCT2, pharmacogenetics, population PK/PD modeling

## Abstract

**Introduction:**

Gabapentin (GBP) is commonly used for chronic neuropathic pain, yet its therapeutic response varies widely across individuals. As a substrate of the organic cation transporter 2 (OCT2), encoded by the *SLC22A2* gene, GBP’s penetration into the central nervous system (CNS) may be influenced by genetic variability. This study aimed to characterize the impact of *SLC22A2* c.808G>T polymorphism on GBP pharmacokinetics (PK) and pharmacodynamics (PD) and inform genotype-guided dosing strategies.

**Methods:**

Data from two clinical studies (n = 94) were pooled, including single and multiple oral dose regimens of GBP. Population PK/PD modeling was performed using nonlinear mixed-effects modeling.

**Results:**

A two-compartment PK model with first-order absorption and linear elimination best described GBP disposition, with estimated apparent clearance (CL/F) significantly influenced by renal function (eGFR). Pain scores revealed delayed pain relief relative to peak plasma levels, requiring an effect compartment to link PK to an Imax model. The *SLC22A2* c.808G>T (OCT2) variant was associated with a 10-fold reduction in the influx rate constant (ke_1_) to the effect site, suggesting impaired CNS drug delivery. Simulations demonstrated that GT carriers experienced markedly reduced pain relief, even at the maximum approved doses, compared to GG homozygotes. Renal impairment increased systemic exposure but did not alter CNS penetration.

**Conclusion:**

These findings highlight the importance of the OCT2 genotype in modulating GBP’s analgesic efficacy. Incorporating transporter pharmacogenetics into PK/PD models may enhance individualized therapy for neuropathic pain, particularly in identifying poor responders who may benefit from alternative dosing or adjunct treatments.

## 1 Introduction

Gabapentin (GBP), initially approved for epilepsy and post-herpetic neuralgia, is widely prescribed for the management of different forms of neuropathic pain (Neurontin, 2022; Wallach and Ross, 2018). Its primary mechanism of action involves binding to the α2δ-1 subunit of voltage-gated calcium channels in presynaptic neurons, reducing calcium influx, and excitatory neurotransmitter release. Recent studies indicate that α2δ-1 also interacts with NMDA receptor (Chen et al., 2014), promoting their synaptic trafficking in neuropathic pain states; GBP mitigates this by inhibiting α2δ-1–NMDA receptor complex formation and trafficking. Additional modulatory effects on inflammatory pathways by decreasing the expression of TLR4 in the dorsal root ganglion (Jin et al., 2022). This interaction occurs at multiple levels of the nervous system, including dorsal root ganglia, spinal cord dorsal horn neurons, and supraspinal regions. By reducing calcium influx during neuronal depolarization, GBP inhibits the release of excitatory neurotransmitters, including glutamate, norepinephrine, and substance P, contributing to decreased neuronal excitability and analgesic efficacy (Fink et al., 2000).

Despite its widespread use, GBP dosing for neuropathic pain remains largely empirical with standard regimens typically ranging from 900 to 3,600 mg/day, titrated based on patient tolerance, response and renal function (Neurontin, 2022; Backonja and Glanzman, 2003). However, clinical failure rates remain substantial, with up to 40%–60% of patients discontinuing therapy due to lack of efficacy or intolerable side effects (Moore et al., 2014; Meaadi et al., 2023). This variability in therapeutic outcomes highlights a critical gap in our understanding of the factors influencing GBP response. Current dosing strategies do not account for interindividual differences in drug absorption, distribution, central nervous system (CNS) penetration, nor do they consider potential pharmacogenetic influences. As such, there is a growing need to refine GBP therapy through a more personalized approach.

GBP is absorbed via an active transport system in the intestine, with minimal impact of food on its bioavailability. Less than 3% of the drug is protein-bound in plasma (Neurontin, 2022; Beydoun et al., 1995). It is not metabolized and is primarily eliminated unchanged via renal excretion, with a half-life of 5–8 h (Neurontin, 2022). In both adult and pediatric populations, renal function is the primary determinant of GBP clearance, necessitating dose adjustments in patients with impaired renal function (Neurontin, 2022; Beydoun et al., 1995). The human Solute Carrier 22 (SLC22) family, also known as the organic ion transporter family, plays a central role in the transport of a wide array of small molecules, including endogenous metabolites, signaling molecules, environmental toxins, and many drugs. It includes the organic anion transporters (OATs; e.g., *SLC22A6/A8*), organic cation transporters (OCTs; *SLC22A1–A3*), and the OCTN subgroup (*SLC22A4/A5*). Organic cation transporters, specifically OCT2 and OCTN1 have been implicated in the renal elimination of GBP (Urban et al., 2008; Lozano et al., 2018). OCT2, encoded by the *SLC22A2* gene, is highly expressed in renal proximal tubule cells and facilitates the active tubular secretion of cationic compounds, including GBP (Lozano et al., 2018). Genetic variation in *SLC22A2* has been associated with interindividual differences in drug disposition, with single-nucleotide polymorphisms (SNPs) identified that may alter transporter function and, consequently, drug pharmacokinetics (Leabman et al., 2002). OCT2 is also expressed in the brain micro vessel endothelial cells, suggesting a possible role in blood–brain barrier (BBB) penetration (Bacq et al., 2012). Furthermore, OCT2 activity has been linked to the modulation of brain neurotransmitter levels (Bacq et al., 2012), raising the possibility of its involvement in central nervous system (CNS) drug delivery and pharmacodynamic effects. Clinical and *in vitro* studies have indicated that OCTN1 facilitates active tubular secretion of GBP. OCTN1, encoded by *SLC22A4*, is expressed in a variety of human tissues, with particularly high levels in the kidney (proximal tubule epithelial cells), intestine (enterocytes), bone marrow/immune system (myeloid/erythroid cells) (Kobayashi et al., 2004), airway (epithelial cells) (Berg et al., 2018), brain endothelium (brain micro vessel endothelial cells) (Betterton et al., 2021). In clinical pharmacology, OCTN1 plays a role in the renal secretion of certain drugs and may influence drug absorption and disposition, especially for substrates that rely on active transport mechanisms. Although the common OCTN1 L503F variant reduces transporter activity (Taubert et al., 2005), the renal clearance of GBP approximates the glomerular filtration rate (Neurontin, 2022). This indicates that passive filtration is the predominant mechanism for gabapentin’s renal elimination, with active tubular secretion by transporters like OCT2 and OCTN1 likely playing a minor role in GBP clearance.

Despite its mechanistic relevance, the clinical impact of *SLC22A2 or SLC22A4* gene variations on GBP analgesic response remains incompletely understood. While renal function is a primary determinant of drug clearance, it represents only one aspect of the drug’s disposition. Target site exposure, particularly in the CNS, is another important aspect to be considered when attempting to appropriately interpret factors impacting therapeutic outcomes. Evidence for a significant effect of the *SLC22A2* c.808G>T variant has been limited, partly due to its low minor allele frequency and insufficient statistical power. To overcome these limitations, we are integrating data from both single-dose (Costa et al., 2020; Costa et al., 2021) and multiple-dose (Yamamoto et al., 2019) studies in patients with chronic neuropathic pain to determine if OCT2 primarily affects GBP therapeutic outcomes via alteration of renal clearance and/or CNS target site exposure. The findings may support the identification of individuals at increased risk of suboptimal treatment outcomes and inform the potential utility of OCT2 genotyping in personalizing GBP therapy.

## 2 Materials and methods

### 2.1 Study population

This analysis included data pooled from two clinical studies involving a total of 94 patients with chronic neuropathic pain who received either single (n = 29) and multiple doses (n = 65) of GBP. All participants provided written informed consent, and both studies were approved by the local ethics committee at the Clinical Hospital of the University of Sao Paulo, Brazil, and deemed as exempt by the University of Florida (IRB202500636).

In Study 1, patients with moderate to severe neuropathic pain [pain scores >4 on a Visual Analog Scale (VAS)] were enrolled (Costa et al., 2021). The underlying causes included diabetic neuropathy or compressive etiologies such as cervical or lumbar disc herniation. All subjects received a single oral immediate-release (IR) formulation (capsules) of 300 mg GBP. PK in plasma was assessed using a rich sampling protocol, and pain intensity was measured concurrently using a VAS (Costa et al., 2021). In Study 2, 65 patients with chronic neuropathic pain receiving multiple oral doses of GBP IR formulation, ranging from 600–3,600 mg/day, were investigated. PK data were collected using a sparse sampling approach (Yamamoto et al., 2019). Demographic, clinical, and PK data from both studies were combined. GBP concentrations in plasma were determined using a validated bioanalytical method, which was linear across the range of 0.2─14 μg/mL, with LLOQ defined at 0.2 μg/mL. All patients were investigated for *SLC22A2* c.808G>T and *SCL22A4* c.1507C>T gene polymorphisms using TaqMan™ genotyping assays, as previously reported (Yamamoto et al., 2019). These SNPs are in genes encoding the organic cation transporters OCT2 and OCTN1, respectively, which are involved in renal and tissue uptake of various drugs.

The average age of participants was 53 ± 11 years, with a slightly higher proportion of women (n = 53) than men (n = 41). Mean body weight was 81.97 ± 17.34 kg and a mean BMI of 29.9 ± 6.15 kg/m^2^, indicating that most study participants were either overweight or obese. Renal function was assessed using mean serum creatinine levels of 0.92 ± 0.41 mg/dL and the mean eGFR, estimated using the 2021 CKD-EPI Equation (Inker et al., 2021), was 90.29 ± 22.79 mL/min/1.73 m^2^. More than half of the participants (52%) had normal or high kidney function (eGFR ≥90 mL/min/1.73 m^2^), 23% had mildly decreased renal function (60–89 mL/min/1.73 m^2^), 12% had mild-to-severe renal impairment (30–59 mL/min/1.73 m^2^), 1% had severe renal impairment (15–29 mL/min/1.73 m^2^), and 12% of participants had missing serum creatinine data (Supplementary Table S1).

Genetic data were available for two *SLC22A2* and *SCL22A4* major gene variations (Supplementary Table S1). For *SLC22A2* c.808 G>T, most participants carried the GG genotype (n = 79), with 15 individuals heterozygous for GT. The overall allelic frequency was 92% for the G allele and 8% for the T allele, which was consistent with the frequency reported in the Latin American population (G = 87–94% and T = 6–13%) (National Library of Medicine, 2024a). For *SLC22A4* c.1507 C>T, genotype distribution was more balanced: 42 participants had the CC genotype, 39 were heterozygous (CT), and 13 were homozygous for the TT variant. The allelic frequency in our study (65% for the C allele and 35% for the T allele) was aligned with reference values from the Latin American population (G = 69–76% and T = 31–24%) (National Library of Medicine, 2024b). Overall, the distribution of both polymorphisms was consistent with the Hardy-Weinberg equilibrium (Χ^2^_*SLC22A2*_ = 0.71 and p = 0.40; Χ^2^_*SLC22A4*_ = 0.65 and p = 0.42). This genetic data provides insight into the variability in transporter activity that may influence drug disposition in the studied population.

### 2.2 Population PK/PD analysis

#### 2.2.1 Population pharmacokinetic analysis

The population PK/PD model was developed in a stepwise fashion, starting with the population PK model, which was subsequently used to inform the PD component by fixing the estimated PK parameters during PD model development. The population PK model was developed using concentration-time data from 568 plasma samples which were analyzed using a nonlinear mixed-effect modeling approach and SAEM algorithm as implemented in Monolix 2024R1 Simulations Plus (United States), 2024a. Plasma concentration data falling below the lower limit of quantification (LLOQ) were treated as left-censored data, with a finite censoring interval set between zero and the LLOQ, indicating that the true value lies within this range. These censored observations accounted for only 9.15% of the total dataset. Once the structural and residual error model was established, demographic and clinical variables including sex, age, body weight, body mass index, serum creatinine, estimated glomerular filtration rate (eGFR), OCT2, and OCTN1 genotypes, were evaluated as potential covariates. Initial covariate screening involved Spearman correlation for continuous variables and ANOVA for categorical variables. Visual diagnostics included scatter plots for continuous covariates and box plots for categorical covariates.

For continuous covariates, the power function model was used, defined by the following equation:

$${\theta_{i}=\theta }_{pop\;}\times {\left(\frac{covariate}{weighted\;mean}\right)}^{\beta }$$

Where *θ*_*i*_ defines the individual parameter, *θ*_*pop*_ is the typical value in the population, *β* is the power coefficient describing the covariate-parameter relationship. A weighted mean of 84.85 mL/min/1.73 m^2^ was used as a reference for eGFR. A weighted mean is an average where each value is multiplied by its weight (number of observations for each individual) and divided by the sum of the weights (total number of observations). Missing continuous covariates values were imputed using the median.

For categorial variables, such as genotypes, a proportional linear model was used, as represented by the following equation.

$${\theta_{i}=\theta }_{pop}\times e^{\left(\beta \times covariate\right)\;}$$

Where *θ*_*i*_ represents the individual parameter, *θ*_*pop*_ is the typical value for reference group, *β* defines the covariate effect coefficient for the other categories, assuming a lognormal distribution. For *SLC22A2* c.808G>T genotype, individuals with the GG genotype were designated as the reference group (coded as 0), and those with the GT genotype as 1. For the *SLC22A4* c.1507 C>T genotype, individuals with the CC, CT and TT genotypes were coded as 0 (reference), 1 and 2, respectively. Covariate selection followed a stepwise approach, combining forward inclusion and backward elimination.

Nested models were compared using the −2 x log-likelihood (-2LL), assuming a chi-squared distribution for the difference in the objective function value (ΔOFV). A drop of 3.84 units per degree of freedom was considered significant for forward inclusion (p < 0.05), and 6.63 units per degree of freedom for backward deletion (p < 0.01). All PK parameters were initially assumed to be log-normally distributed and include random effects (*η*); however, the need for random effects and the distributional assumptions were reassessed during model development. The model’s predictive performance was assessed using visual predictive checks (VPC).

#### 2.2.2 Population PK/PD model

Once the population PK model was established and verified, drug exposure was linked to response using an inhibitory effect model (I_max_) through the development of a PK/PD model. Pain intensity was assessed using VAS, varying from 0 (no pain) to 10 (worst possible pain). Given that pain scores were measured in a numerical rating scale and intervals between values are equal, it was considered a continuous variable in the PD model. A total of 392 pain score observations were used for model development.

As GBP exerts its analgesic effect through reversible binding to α2δ-1 subunit of voltage-gated calcium channels in presynaptic neurons, thereby reducing neuronal excitability, both sigmoid and non-sigmoid I_max_ models were evaluated to describe the concentration-response relationship. To account for the observed temporal delay between plasma concentrations and response (Supplementary Figure S1), an effect compartment was incorporated to link the PK and PD models. Various model configurations were tested, including models with identical rate constants for drug transfer in and out of the effect compartment (ke_1_ = ke_2_), as well as models with distinct rate constants (ke_1_ ≠ ke_2_). All PK/PD parameters were assumed to follow log-normal distributions, except for the baseline pain score (E_0_), which was constrained within 0 and 10, using a logit distribution. Given that both OCT2 and OCTN1 are expressed in endothelial cells of the nervous system, potentially influencing drug uptake at the target site (Pochini et al., 2022; Dos Santo et al., 2014), a covariate analysis was conducted on the PD parameters to evaluate their potential role in GBP’s response. Parameter uncertainty was assessed using non-parametric bootstrap resampling (n = 500 replicates) implemented in Monolix 2024R1, yielding median estimates and 2.5th and 97.5th percentiles for all model parameters. For runs with failed convergence, up to 20 reattempts were allowed, ensuring a minimum convergence rate of 96% for the final model.

### 2.3 Clinical trial simulations

The PK/PD model was employed in clinical trial simulations to evaluate the impact of the covariates on GBP’s analgesic response using Simulx 2024R1 Simulations Plus (United States), 2024b. A 5-week simulation period was used to ensure PD steady-state conditions. Drug exposure was assessed using the area under the plasma concentration-time curve during a dosage interval (AUC_τ_) and maximum concentration (C_max_). Median values and 10th-90th percentiles were computed using R (version 4.5.1) for comparison of dosing regimens. For visualization purposes, pain scores were further converted into pain attenuation (% reduction from baseline). For each scenario, the proportion of subjects experiencing substantial pain relief (≥50% reduction from baseline) and moderate pain relief (30%–50% reduction) were recorded. Pain relief was evaluated at the time of maximum pain attenuation and using the area under the effect curve during a dosage interval (AUEC_τ_). Areas under the exposure and response curves were calculated using the trapezoid method in R (version 4.5.1, package “pracma_2.4.4”). The duration of the response was assessed using the probability of target attainment (PTA), defined as the proportion of subjects achieving ≥50% pain relief for at least 80% of the dosing interval. A PTA ≥80% was considered indicative of sustained analgesic efficacy.

## 3 Results

### 3.1 Population PK/PD model

**FIGURE 1:**
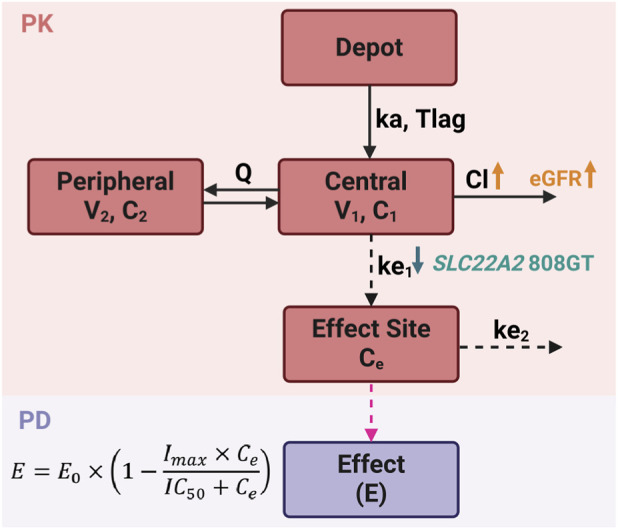
Schematic representation of the final PK/PD model structure. The PK model consists of a depot compartment with first-order absorption and lag time (k_a_: absorption rate constant; t_lag_: lag time); two-compartments (V_1_: volume of distribution of central compartment; V_2_: volume of distribution of peripheral compartment) connected through intercompartmental clearance (Q); and linear elimination from the central compartment (CL: clearance). The covariate effect of eGFR on CL and *SLC22A2* c.808G>T genotype on ke_1_ is represented by the orange and green arrows. The PD component is represented by an inhibitory effect model (I_max_), where the effect is driven by the drug concentration (C_e_) in the effect compartment, which is linked to the central compartment through a first-order rate constant (ke_1_) and a rate constant coming out of the effect compartment (ke_2_). The I_max_ model described the drug exposure-effect relationship.

The population PK model for GBP was best described by a two-compartment structure with first-order absorption, a lag time (t_lag_), and linear elimination (Figure 1). The estimated t_lag_ was short (0.34 h), and the absorption rate constant (k_a_) was 0.14 h^-1^. Apparent clearance (CL/F) was estimated at 10.16 L/h and was significantly influenced by renal function, as captured by the covariate effect of eGFR on CL/F, with a coefficient of 1.34. This confirms the expected dependency of GBP clearance on renal function. Based on this model, patients with mean eGFR values of 100 (normal renal function), 75 (mild renal impairment), and 45 mL/min/1.73 m^2^ (mild-severe renal impairment) are predicted to have typical CL/F values of 12.66, 8.61, and 4.34 L/h, respectively. A constant residual error model best described the unexplained residual variability in the PK model (Table 1).

**TABLE 1:** Parameter estimates for the population joint PK/PD model of gabapentin in patients with neuropathic pain.

Parameter	Final estimate (%RSE)	Bootstrap median (95% CI)
Fixed effects		
t_lag_ (h)	0.34 (8.03)	0.32 (0.23–0.42)
k_a_ (h^-1^)	0.14 (7.04)	0.15 (0.13–0.19)
CL/F (L/h)	10.16 (12.15)	10.06 (6.57–13.40)
logt (eGFR/84.85) on CL/F	1.34 (24.38)	1.35 (0.56–2.20)
V_1_/F (L)	18.16 (15.13)	20.98 (14.43–30.72)
Q (L/h)	6.58 (18.68)	7.25 (3.82–11.49)
V_2_/F (L)	357.67 (31.27)	335.79 (149.49–854.09)
ke_1_ (h^-1^)	0.53 (55.76)	0.47 (0.14–1.38)
OCT2 on ke_1_	−2.44 (41.81)	−2.42 (−4.81–−0.80)
E_0_	7.31 (5.86)	7.27 (6.01–8.23)
IC_50_ (ng/mL)	263.11 (fixed)	-
ke_2_ (h^-1^)	1 (fixed)	-
I_max_	1 (fixed)	-
Random effects		
ω_Tlag_	0.52 (14.23)	0.56 (0.37–0.81)
ω_ka_	0.15 (45.06)	0.11 (0.037–0.29)
ω_CL_	0.46 (11.17)	0.46 (0.32–0.61)
ω_V1_	0.54 (18.34)	0.53 (0.30–0.70)
ω_Q_	0.64 (16.19)	0.57 (0.34–0.84)
ω_ke1_	2.11 (14.59)	2.02 (0.91–3.19)
ω_E0_	0.98 (17.26)	1.03 (0.47–1.76)
Residual error model for PK		
Additive (a)	0.19 (4.44)	0.18 (0.13–0.24)
Residual error model for PD		
Additive (a)	0.52 (5.81)	0.53 (0.36–0.97)
Proportional (b)	0.30 (9.63)	0.29 (0-0.48)

%RSE: relative standard error; CI: confidence interval; CL/F: apparent clearance; E_0_: baseline effect; eGFR: estimated glomerular filtration rate; IC_50_: half-maximal inhibitory concentration; I_max_: full inhibition (fixed to 1); k_a_: absorption rate constant; ke_1_: first order rate constant coming in the effect compartment; ke_2_: rate constant coming out of the effect compartment; Q: intercompartmental clearance; t_lag_: lag time; V_1_/F: apparent volume of distribution of central compartment; V_2_/F: apparent volume of distribution of peripheral compartment; ω: interindividual variability, expressed as standard deviation of the random effects.

An I_max_ model with effect compartment and full inhibition (I_max_ set to 1) was used to describe the relationship between GBP exposure and pain attenuation, as measured by VAS scores. The effect compartment was linked to the central compartment using first-order rate constants ke_1_ and ke_2_ to describe the rate constants into and out of the effect compartment, respectively. Given that the primary goal of the study was to investigate the role of drug transporters which could be linked to GBP’s transfer to the effect compartment, but not to its pharmacodynamic potency, the half-maximal inhibitory concentration (IC_50_) was fixed to reduce parameter uncertainty and improve model identifiability. In the final modeling step, the PK parameters were unfixed, and all PK/PD parameters were estimated simultaneously. The resulting joint PK/PD model was subsequently used to perform simulations.

The *SLC22A2* c.808G>T genotype was a significant covariate on ke_1_ parameter. Inclusion of this covariate led to a decrease in the OFV of 32.94, indicating improved model fit. Individuals carrying the GT genotype exhibited a 10-fold decrease in the estimated influx rate, with ke_1_ values of 0.53 h^-1^ for GG and 0.05 h^-1^ for GT genotypes. Parameter estimates were consistent with the bootstrap medians and fell within the 95% bootstrap confidence intervals, supporting the robustness of the model (Table 1). Predicted GBP plasma concentrations showed strong agreement with observed data, with no evidence of systematic bias (Supplementary Figures S2, S3).

### 3.2 Simulations

The impact of covariates was evaluated through simulations of multiple dose regimens, following the label dosing recommendation for the IR formulation based on renal function (Neurontin, 2022). Virtual cohorts of 200 subjects were generated with varying degrees of renal function categorized as follows: normal or high (Stage 1, eGFR from 90–120 mL/min/1.73 m^2^), mildly decreased (Stage 2, eGFR: 60–89 mL/min/1.73 m^2^), mild to severe decreased (Stage 3, eGFR: 30–59 mL/min/1.73 m^2^) and severe decreased (Stage 4, eGFR: 15–29 mL/min/1.73 m^2^). Within each category, eGFR values were sampled from a uniform distribution. The effect of the *SLC22A2* c.808G>T genotype was simulated, assuming a frequency of 100% for each genotype within each renal function subgroup. Maintenance doses regimens were simulated at increasing doses with different dose interval based on renal function: a) Stages 1 and 2: 300, 400, 600, 900, and 1200 mg three times daily (TID), b) Stage 3: 200, 300, 400, and 700 mg twice daily (BID), c) Stage 4: 200, 300, 400 and 700 mg once daily (QD).

**FIGURE 2:**
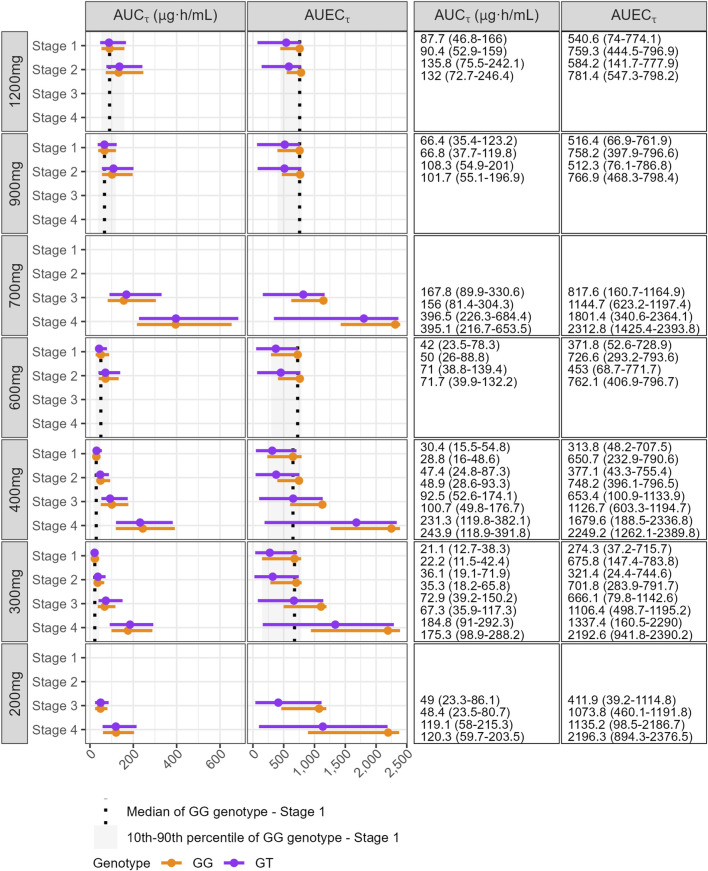
Effect of *SLC22A2* c.808G>T genotype and renal function stage on the exposure (AUC_τ_) and overall pain relief (AUEC_τ_) at steady-state (5 weeks). Simulations of multiple dosing regimens with doses ranging from 200 mg up to 1,200 mg administered three times daily (TID) for Stages 1 and 2, twice daily (BID) for Stage 3, and once daily (QD) for Stage 4. The group with SLC22A2 GG genotype and normal or high renal function (Stage 1), represented as median (dotted line) and 10-90th percentiles (shaded area), was used as reference group. The data is represented as median (solid circle) and 10-90th percentiles (error bar), with homozygous wild-type (GG) and heterozygous (GT) genotypes in orange and purple, respectively. Renal function stages: Stage 1 – normal or high renal function (eGFR ≥90 mL/min/1.73 m^2^); Stage 2 – mild decreased (60–89 mL/min/1.73 m^2^); Stage 3 – moderate to severe decreased (30–59 mL/min/1.73 m^2^); Stage 4 – severe decreased (15–29 mL/min/1.73 m^2^). AUC_τ_: area under the plasma concentration-time curve during a dosage interval; AUEC_τ_: area under the effect-time curve during a dosage interval.

As anticipated, the *SLC22A2* c.808G>T genotype did not affect systemic exposure (AUC_τ_ and C_max_) when comparing individuals within the same dose regimen and renal function. However, pain attenuation (AUEC_τ_ and maximum pain attenuation) was reduced in heterozygous (GT) individuals. This reduction is attributed to the genotype effect on ke_1_, where reduced transporter activity slows the rate of drug entry into the target site (Figure 2; Supplementary Figure S4).

**FIGURE 3:**
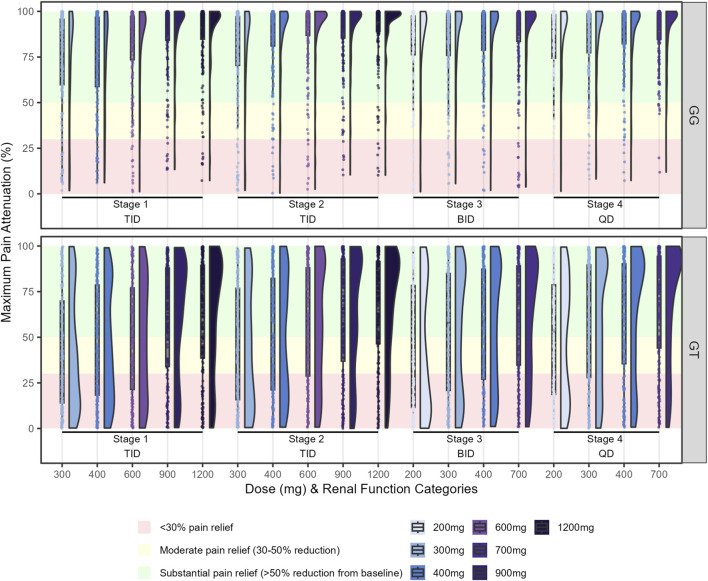
Distribution of maximum pain attenuation response at steady-state. Density plots illustrate the distribution of individual responses for each genotype and dosing regimen. Shaded background areas correspond to predefined response categories: <30% pain relief (red), moderate pain relief (30%–50% reduction from the baseline, yellow), and substantial pain relief (≥50% reduction from the baseline, green). Density curves and box-plots summarize the overall distribution of responses and points represent the individual data. *SLC22A2* c.808 genotype: GG (homozygous wild-type) and GT (heterozygous). TID: three times daily, BID: twice daily, QD: once daily. Renal function stages: Stage 1 – normal or high renal function (eGFR ≥90 mL/min/1.73 m^2^); Stage 2 – mild decreased (60–89 mL/min/1.73 m^2^); Stage 3 – moderate to severe decreased (30–59 mL/min/1.73 m^2^); Stage 4 – severe decreased (15–29 mL/min/1.73 m^2^).

**FIGURE 4:**
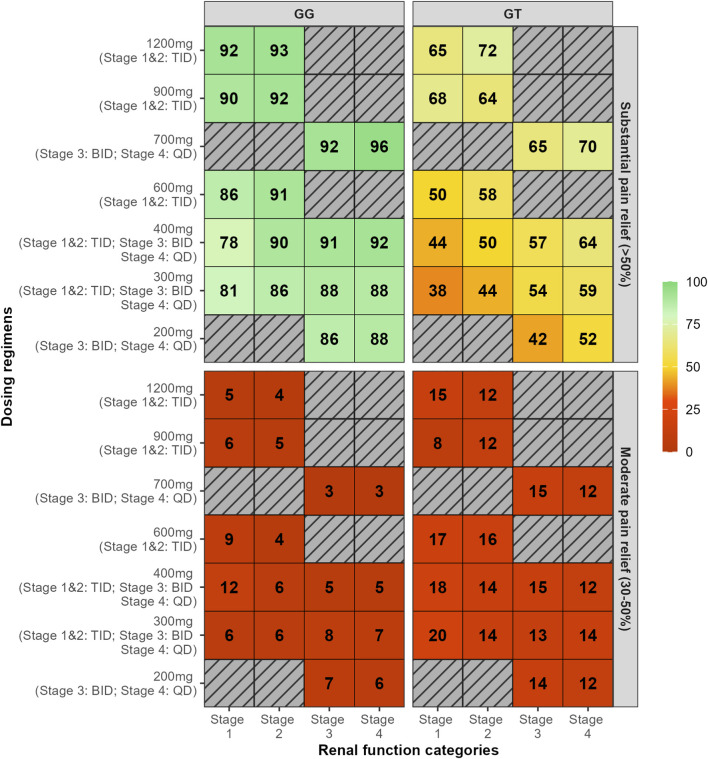
The heat map represents the percentage of subjects for each simulation who achieved the maximum pain attenuation above 50% (substantial pain relief) and between 30% and 50% (moderate pain relief). Simulations of multiple dose regimen with dose ranging from 200 mg up to 1,200 mg administered three times daily (TID, Stage 1 and 2), twice daily (BID, Stage 3), and once daily (QD, Stage 4). Heat map colors indicate the threshold: red for values below 30% and green for values equal to or above 80%. SLC22A2 c.808 genotype: GG (homozygous wild-type) and GT (heterozygous). Renal function stages: Stage 1 – normal or high renal function (eGFR >90 mL/min/1.73 m^2^); Stage 2 – mild decreased (60–89 mL/min/1.73 m^2^); Stage 3 – moderate to severe decreased (30–59 mL/min/1.73 m^2^); Stage 4 – severe decreased (15–29 mL/min/1.73 m^2^). Gray-striped dosing regimens were not simulated because they are not recommended in the approved label.

In Figure 3, maximum pain attenuation (0–100) is shown across dose levels within renal function Stages 1–4, with dosing frequencies TID (Stages 1–2), BID (Stage 3), and QD (Stage 4). Density and box plots depict the distribution at each dose (width ∝ data density), with overlaid points indicating individual observations, comparing genotypes GG (top) and GT (bottom). The plots revealed distinct response patterns by genotypes. GT carriers consistently exhibited lower maximum pain attenuation values, with a bimodal distribution particularly evident at lower doses. Although higher doses increased the maximum pain attenuation, responses among GT genotype subjects remained highly variable, whereas GG homozygotes showed more consistent and substantial pain relief (Figure 3). We summarized model-based probabilities of analgesic response across dose–renal function groups and genotypes. Heatmaps are stratified by genotype (GG, left panel; GT, right panel) and by outcome: substantial pain relief (>50%) in the upper block and moderate pain relief (30%–50%) in the lower block. Rows correspond to dosing regimens (dose shown at left; the applicable frequency by renal stage—TID in Stages 1–2, BID in Stage 3, QD in Stage 4—is indicated in parentheses). Columns denote renal function categories (Stages 1–4). Each cell reports the predicted probability (%) for the specified outcome, and color encodes the magnitude (red = low, green = high). We simulated dosing regimens indicated in the label, regimens not evaluated were represented as hatched cells. Across all simulated dosing regimens, 78%–96% of GG subjects achieved a maximum pain attenuation above 50%, with only 1%–13% showing minimal relief (maximum pain attenuation <30%). In contrast, 16%–44% of GT subjects did not achieve pain relief, highlighting the greater variability in response within this genotype (Figure 4).

**FIGURE 5:**
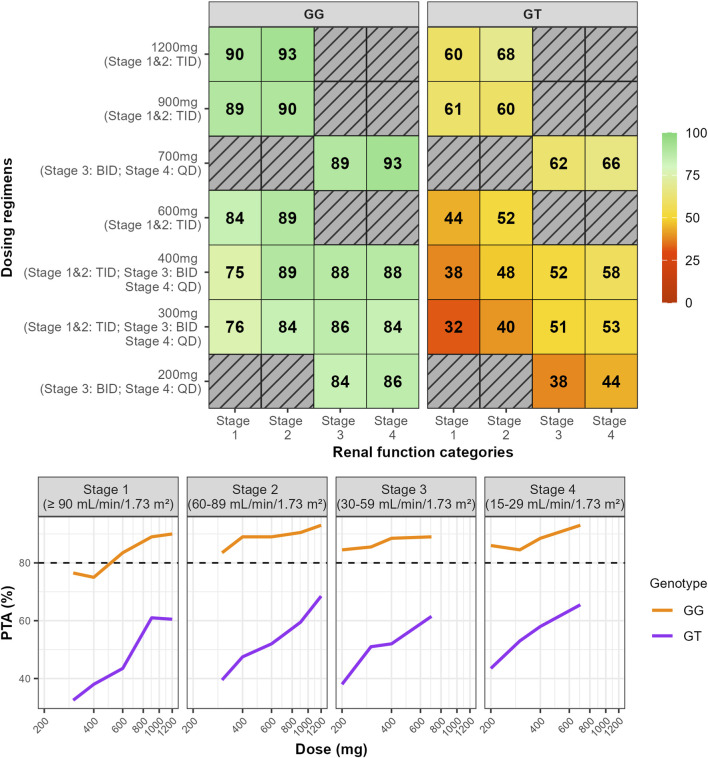
Percentage of subjects who achieved pain relief above 50% for at least 80% of the time interval, assuming the last dose administered at steady-state. Dose intervals were three times daily (TID), twice daily (BID), and once daily (QD) for Stage 1 and 2, Stage 3, and Stage 4, respectively. Heat map colors indicate the threshold: red for values below 80% and green for values equal to or above 80%. *SLC22A2* c.808G>T genotype: GG (homozygous wild-type) and GT (heterozygous). Renal function stages: Stage 1 – normal or high renal function (eGFR >90 mL/min/1.73 m^2^); Stage 2 – mild decreased (60–89 mL/min/1.73 m^2^); Stage 3 – moderate to severe decreased (30–59 mL/min/1.73 m^2^); Stage 4 – severe decreased (15–29 mL/min/1.73 m^2^). Gray-striped dosing regimens were not simulated because they are not recommended in the approved label.

**FIGURE 6:**
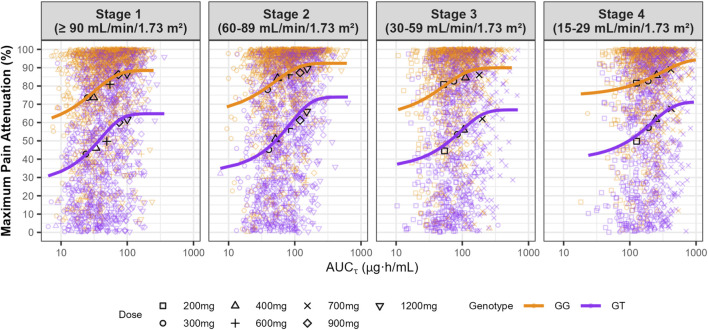
Exposure-response curve for GBP in patients with chronic neuropathic pain at different renal function stages. Black symbols correspond to the mean value for each dosing regimen. Dose intervals were three times daily (TID), twice daily (BID), and once daily (QD) for Stage 1 and 2, Stage 3, and Stage 4, respectively. *SLC22A2* c.808G>T homozygous wild-type (GG) and heterozygous (GT) genotype are represented by orange and purple colors, respectively.

We quantified the PTA across simulated dose levels (x-axis) and renal function categories (four panels). In patients with normal renal function, simulations predict that 76% of GG carriers achieve substantial pain relief (≥50% reduction) for at least 80% of the dosing interval with 300 mg TID. This proportion increased to 84% with 600 mg TID (Figure 5). In contrast, only 32% of GT carriers with normal renal function are expected to maintain substantial pain relief with 300 mg TID. Even with the maximum approved dose of 1,200 mg TID (3,600 mg/daily), only 60% reaches substantial pain relief for ≥80% of the dosing interval (Figure 5). These findings suggest that a high proportion of GT carriers are poor responders to GBP, highlighting the need for optimized treatment and dosing strategies. Additionally, the exposure-response curves demonstrated that the GT genotype consistently shows lower maximum pain attenuation at similar AUC levels compared to the GG genotype. However, even at high plasma exposures achieved with the maximum approved doses of 3,600 mg daily, GT carriers did not achieve an average maximum pain attenuation greater than 65% in patients with normal renal function (Figure 6). It is also important to highlight the greater variability among GT carriers at comparable exposure levels (Figure 6)

Decreased renal function reduces GBP clearance, leading to increased plasma exposure. Simulations demonstrated that patients with Stage 2, 3, and 4 renal impairments exhibited approximately 1.6-, 3.0-, and 7.9-fold increases in AUC_τ_, respectively, compared to patients with normal renal function receiving 300 mg at each dosing interval and *SLC22A2* 808 GG genotype. The *SLC22A2* c.808G>T genotype does not impact GBP PK, and as a result, the exposure changes due to renal function are consistent across both wild-type GG and GT carriers (Figure 2). It is important to note that simulations for Stage 3 and 4 renal impairments followed the approved dosing recommendation, using BID and QD dosing intervals. For example, 84% of Stage 3 patients receiving 400 mg/day as 200 mg BID achieved substantial pain relief for ≥80% of the dosing interval. Similarly, 86% of Stage 4 patients receiving 400 mg QD reached the same level of pain relief (Figure 5). Despite reduced dosing in lower renal stages, the exposure-response curves still show meaningful efficacy, indicating that dose adjustments maintain therapeutic levels (Figure 6).

## 4 Discussion

In this study, we aimed to develop an integrated population PK/PD model to simultaneously evaluate the effects of renal function and OCT2 pharmacogenetics on GBP’s analgesic efficacy. The results of our analysis confirmed that renal function plays a key role in GBP elimination, while *SLC22A2* c.808G>T (OCT2) variant was associated with a reduced influx rate constant into the effect compartment, suggesting impaired CNS drug penetration. Simulations based on the developed model provide a framework to guide prescribers in selecting individualized dosing regimens that account for both OCT2 genotype and renal function, using eGFR calculated via the 2021 CKD-EPI equation.

Consistent with previous population PK models of GBP, renal function was found to be a covariate on drug clearance (Costa et al., 2021; Yamamoto et al., 2019; Al-Zubaydi et al., 2024; Ahmed et al., 2017; Carlsson et al., 2009; Tran et al., 2017). Patients with impaired renal function exhibited increased exposure to GBP, underscoring the drug’s reliance on renal elimination. Simulations conducted for Stage 3 and Stage 4 chronic kidney disease (CKD) incorporated BID and QD dosing regimens, respectively, in alignment with label recommendations to avoid unnecessary elevation of GBP plasma concentrations. Our findings demonstrate the significant influence of renal function on analgesic response. Importantly, when dosing intervals were adjusted appropriately—such as 300 mg TID for Stages 1 and 2, BID for Stage 3, and QD for Stage 4—the proportion of 808 GG carriers achieving substantial pain relief remained comparable across groups, varying from 81% to 88%.

OCT2 is a key renal basolateral transporter responsible for the uptake of a wide range of exogenous and endogenous compounds from blood into the proximal tubule cells, thereby facilitating their elimination via the urine. Following uptake into the tubule cells, renal secretion involves subsequent excretion via multidrug and toxin extrusion (MATE1 and MATE2-K) transporters, P-glycoprotein (P-gp), or the multidrug resistance protein (MRP) from epithelial cells into the tubular lumen (Asano et al., 2025). Since OCT2 and MATEs overlap for multiple substrate specificities and their coordinated activity is required for renal excretion, substrates for OCT2/MATE, such as cationic drugs metformin, cisplatin and morphine, are secreted into urine via OCT2/MATEs, whose activity impacts both efficacy and toxicity profiles (Asano et al., 2025; Koepsell, 2020; Yang et al., 2021). Interactions with OCT2 using cimetidine and tyrosine kinase inhibitors can reduce renal clearance, leading to increased systemic exposure and potential toxicity (Sprowl et al., 2013). GBP’s renal clearance is 5.3 L/h (Costa et al., 2020). Once the drug is poorly bound to plasma proteins, with a fraction unbound (fu) of 0.97, the observed renal clearance is similar to the product of fu × GFR, suggesting that glomerular filtration is a major component of renal excretion, and tubular secretion and reabsorption do not contribute to excretion through urine. This finding supports the absence of an observable effect of the OCT2 genotype on gabapentin renal clearance in the present study.

OCT2 is also known to modulate brain levels of neurotransmitters, since serotonin (5-HT) and noradrenaline (NE), and tryptophan, a precursor to serotonin (Orrico-Sanchez et al., 2023), are known OCT2 substrates. Its activity has been related to anxiety and depression behavior and treatment (Bacq et al., 2012; Orrico-Sanchez et al., 2023; Mooney et al., 2008; Wultsch et al., 2009). Therefore, reduced activity could theoretically lower neuronal excitability. However, our data suggested the opposite effect: carriers of the reduced-function variant (808 GT) exhibited diminished analgesic response to GBP. This suggests that OCT2-mediated transport, rather than neurotransmitter modulation, plays a more dominant role in GBP’s CNS pharmacodynamics. OCT2 expression has been confirmed on brain capillary endothelial cells, particularly on the luminal surface (Hoshi et al., 2013; Lin et al., 2010). Functional data in rodents show that knockdown of OCT2/OCT1 in isolated brain capillaries reduces uptake of substrates (e.g., MPTP) by up to 90%, indicating active transporter-mediated uptake across the BBB (Lin et al., 2010).

Previous work from our group demonstrated that GBP is a low-apparent-affinity substrate for human OCT2 (hOCT2) in HEK cells, with an IC_50_ of 40.6 μg/mL (Costa et al., 2020). However, low affinity should not be mistaken for a lack of functional relevance, particularly when OCT2 is highly expressed in key tissues such as the brain (Bacq et al., 2012) and dorsal root ganglia (Sprowl et al., 2013). In such contexts, GBP concentrations may reach sufficient levels to engage OCT2-mediated transport, thereby influencing drug distribution. Moreover, the common OCT2 variant 808 G>T (Ala270Ser) has been described either to reduce (Leabman et al., 2002; Kang et al., 2007; Song et al., 2008) or to enhance transporter activity (Chen et al., 2009; Chen et al., 2007). Even for a low-affinity substrate like GBP, reduced OCT2 function in carriers of this variant may alter intracellular drug distribution by limiting or delaying transfer to the site of action in the spinal cord. This is especially relevant given that GBP’s effect compartment is likely the neuronal tissue, where OCT2 is expressed and may play a role in mediating drug uptake. In fact, the *SLC22A2* c.808G>T variant was associated with a reduced ke_1_, indicating slower drug transfer into the effect compartment—a surrogate for CNS exposure. This reduction had a notable impact on pain attenuation response. For example, among patients with normal renal function (eGFR 90–120 mL/min/1.73 m^2^), only 32% of GT carriers achieved substantial pain relief (≥50% reduction from baseline) following 300 mg TID, compared to 76% of GG carriers. These findings highlight the potential utility of preemptive *SLC22A2* genotyping to identify poor responders who may benefit from higher initial doses or adjunct analgesic therapy for pain relief.

Our simulations indicate that carriers of the *SLC22A2* c.808G>T variant are unlikely to achieve substantial pain relief when treated with low GBP doses. For example, among individuals with normal renal function receiving 300 mg TID, only 32% were predicted to respond with substantial analgesia. Increasing the dose to 600 mg TID modestly improved the response rate to 44%. These findings suggest that *SLC22A2* c.808G>T carriers may require higher GBP doses, ranging from 900–1200 mg TID, as tolerated, to enhance the likelihood of therapeutic benefit. Importantly, the greater variability in response among GT carriers at a given exposure level supports the need for individualized dose titration, particularly for this subgroup. It is important to note that our study did not assess GBP tolerability. Therefore, dose escalation in TT carriers should follow a titration regimen, albeit a steeper increase to reach analgesic response. If higher doses are not tolerated, adjunctive therapy should be considered to achieve adequate pain management in this population.

It is important to note that all simulations and analyses in this study were based on data from GBP IR formulations. Dose titration as well as PK and PD profiles of extended-release (ER) GBP may differ significantly due to altered absorption kinetics, reduced peak-to-trough fluctuations, and potentially delayed onset of action. These differences could influence both systemic exposure and CNS penetration, particularly in individuals with altered OCT2 function. While ER formulations may offer improved tolerability and adherence through less frequent dosing, their slower absorption profile could further exacerbate the delayed CNS distribution observed in carriers of the *SLC22A2* c.808G>T variant. Therefore, extrapolation of our findings to ER GBP should be approached with caution, and future studies are warranted to evaluate whether genotype-guided dosing strategies remain applicable across different formulations.

This study has several limitations. First, due to the low frequency of the *SLC22A2* 808G>T in Latin American populations, no homozygous variant (TT) carriers were enrolled. These individuals may exhibit even lower OCT2 activity and poorer CNS penetration of GBP, potentially resulting in further reduced analgesic response. Secondly, the oral bioavailability of GBP is known to be inversely proportional to the dose. GBP is actively absorbed from the small intestine via the L-amino acid transporters (LATs), a saturable mechanism that contributes to its non-linear, dose-dependent pharmacokinetics. However, due to the limited PK data of different dosages in our study, we were unable to identify changes in bioavailability. LAT1 is highly expressed at BBB and in brain tissue, where it facilitates the entry of essential amino acids and amino acid–mimetic drugs, including GBP (Dickens et al., 2013; Kageyama et al., 2000). In a cohort of 392 Pakistani patients with neuropathic pain, the LAT1 (*SLC7A5*) rs4240803 polymorphism was significantly associated with variability in gabapentinoid (GBP/pregabalin) efficacy and adverse effects. Specifically, the GA genotype was linked to poorer analgesic response and a higher incidence of dizziness and somnolence, suggesting that LAT1 genetic variation may influence GBP uptake and clinical outcomes (Shaheen et al., 2021). Beyond this study, there is limited evidence that known LAT1 SNPs affect drug absorption or efficacy. Further investigation into LAT1 genetic variants is warranted to better elucidate their role in interindividual variability in GBP response.

This study highlights the importance of integrating both renal function and OCT2 pharmacogenetics into GBP dosing strategies for patients with chronic neuropathic pain. While GBP clearance is primarily governed by glomerular filtration and unaffected by OCT2 genotype, the *SLC22A2* c.808G>T variant significantly impairs drug transfer to the CNS, reducing analgesic efficacy. GPB toxicity has been documented in patients with CKD or those undergoing hemodialysis, as the drug’s primary elimination route is renal. This toxicity, which is often due to inappropriately high dosages, can manifest as a range of neurotoxic symptoms, including tremors, altered mental status (confusion, somnolence), and, in severe cases, respiratory depression requiring intubation (Jones et al., 2002; Zand et al., 2010). In poor responders with renal impairment, increasing the GBP dosage may not yield additional analgesic benefit but elevates the risk of adverse events. These findings support the potential clinical utility of preemptive genotyping to identify poor responders and guide personalized therapy. Further research is warranted to evaluate the impact of homozygous variant carriers and severe renal impairment on GBP pharmacodynamics.

## Data Availability

The original contributions presented in the study are included in the article/Supplementary Material; further inquiries can be directed to the corresponding authors.
