# Supplementary material for: Gabapentin CNS exposure and analgesic response are modulated by OCT2 genotype in patients with chronic neuropathic pain
Source: Front Pharmacol. 2026 Mar 12;17:1760901. doi: 10.3389/fphar.2026.1760901 (PMC13018124; doi:10.3389/fphar.2026.1760901)
Supplement: Supplementary file 1 [file Supplementaryfile1.docx]

Supplementary Material

Gabapentin CNS Exposure and Analgesic Response are Modulated by OCT2 Genotype in Patients with Chronic Neuropathic Pain

Lina Zhou^1^, Priscila A Yamamoto^1^, Melody Walker^1^, Ana Carolina C Costa^1^, Gabriela R Lauretti^2^, Fabiola Dach^2^, Stephan Schmidt^1*^, Natalia de Moraes^1*^

^1^Center for Pharmacometrics & Systems Pharmacology, College of Pharmacy, University of Florida, Orlando, FL, USA

^2^School of Medicine of Ribeirao Preto, University of Sao Paulo, Brazil

**LIST OF FIGURES AND TABLES**

Supplementary Table 1. Demographic and clinical data

Supplementary Figure 1. Plasma concentration-time and response profiles for individual subjects

Supplementary Figure 2. Goodness of fit plots of the final PKPD model for gabapentin

Supplementary Figure 3. Visual predictive check (VPC) of gabapentin plasma concentration (top) and pain score (bottom)

Supplementary Figure 4. Effect of *SLC22A2* c.808G>T genotype and renal function stage on the maximum concentration (C_max_) and maximum pain attenuation at steady-state (5 weeks)

Supplementary Data 1. Final PK/PD structural model code in Monolix

Supplementary Data 2. Final PK/PD structural model code in Simulx

**Supplementary Table 1.** Demographics and clinical data

| **Variables** | **Study 1 (n=29)** | **Study 2 (n=65)** | **Total (n=94)** |
| --- | --- | --- | --- |
| Age (years) | 51 ± 6 | 54 ± 12 | 53 ± 11 |
| Sex (n) |  |  |  |
| Men  Women | 10  19 | 31  34 | 41  53 |
| Body weight (kg) | 86.79 ± 19.98 | 79.82 ± 15.72 | 81.97 ± 17.34 |
| BMI (kg/m^2^) | 31.85 ± 6.25 | 29.03 ± 5.96 | 29.9 ± 6.15 |
| Serum creatinine (mg/dL)^a^ | 0.92 ± 0.36 | 0.91 ± 0.43 | 0.92 ± 0.41 |
| eGFR (mL/min/1.73 m^2^)^b^ | 88.18 ± 22.99 | 91.43 ± 22.82 | 90.29 ± 22.79 |
| Normal or high (≥ 90 mL/min/1.73 m^2^)  Mild (60-89 mL/min/1.73 m^2^)  Mild-to-severe (30-59 mL/min/1.73 m^2^)  Severe (15-29 mL/min/1.73 m^2^)  Missing | 16  8  5  0  0 | 33  14  6  1  11 | 49  22  11  1  11 |
| *SLC22A2* c.808 G>T |  |  |  |
| *Genotype [n (%)]*  GG  GT | 22 (76%)  7 (24%) | 57 (88%)  8 (12%) | 79 (84%)  15 (16%) |
| *Allelic frequency (%)*  *G*  *T* | 88  12 | 94  6 | 92  8 |
| *SLC22A4* c.1507 C>T |  |  |  |
| *Genotype [n (%)]*  CC  CT  TT | 11 (38%)  13 (45%)  5 (17%) | 31 (48%)  26 (40%)  8 (12%) | 42 (45%)  39 (41%)  13 (14%) |
| *Allelic frequency (%)*  *C*  *T* | 60  40 | 68  32 | 65  35 |

Data presented as mean ± SD or frequency (%). BMI: body mass index; eGFR: estimated glomerular filtration.

^a^In Study 2, serum creatinine data were available for 54 subjects.

^b^Chronic kidney disease (CKD) stages. eGFR estimated using CKD-EPI 2021 equation (21).


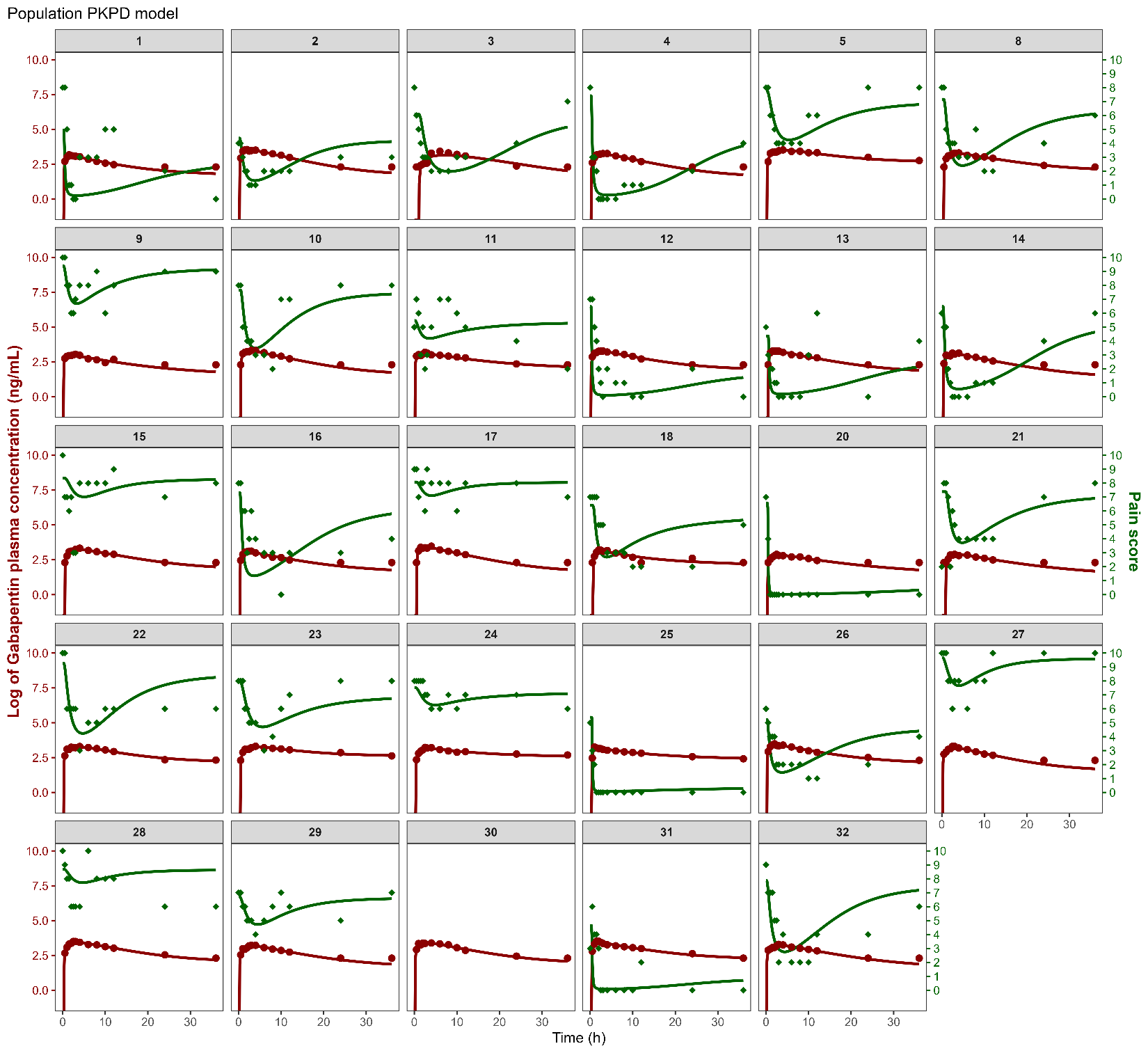


**Supplementary Figure 1.** Plasma concentration–time and response profiles for individual subjects. Individual predicted plasma concentrations and pain scores are represented by red and green lines, respectively, while individual observed data are represented by circles


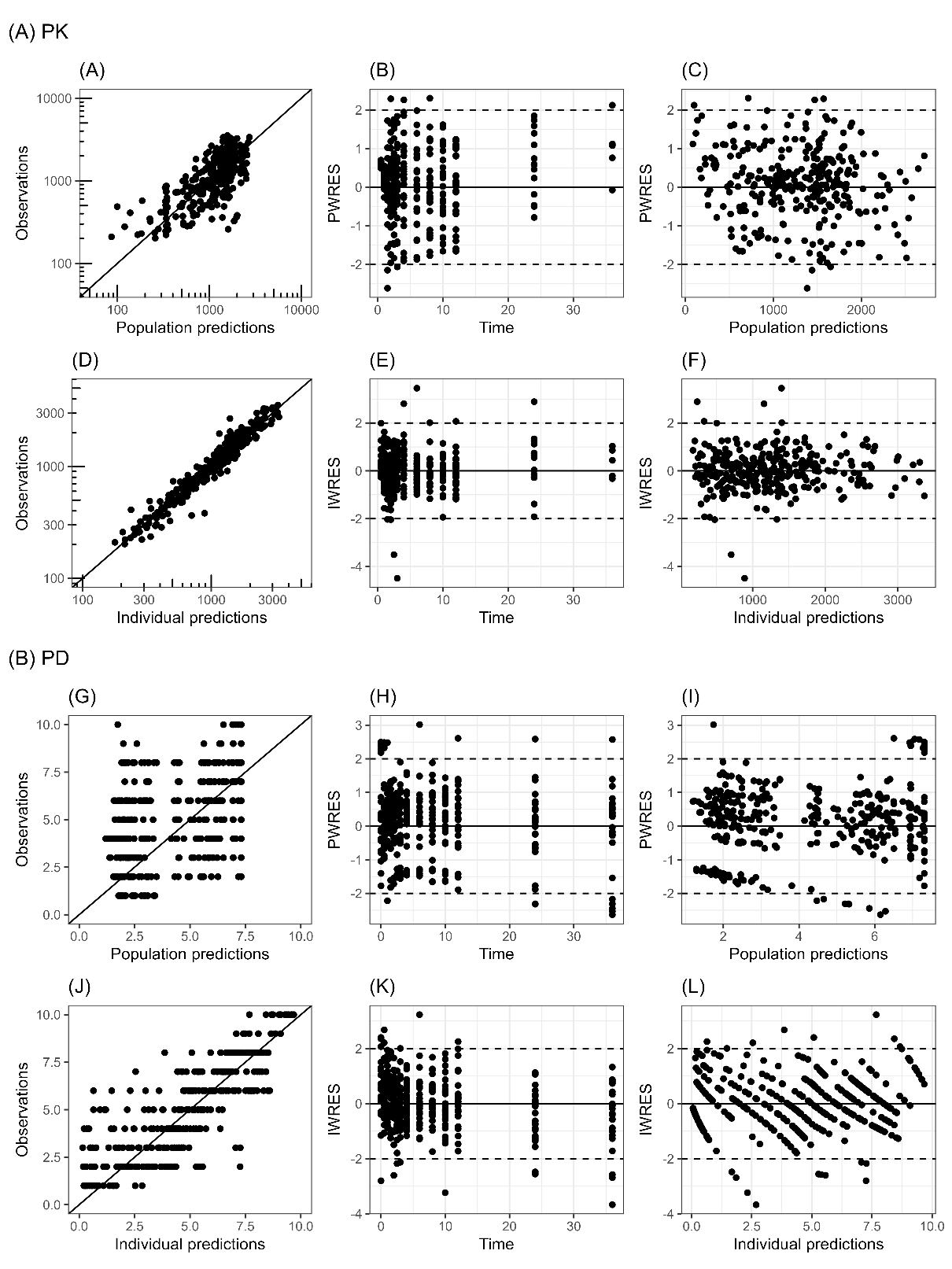


**Supplementary Figure 2.** Goodness of fit plots of the final PKPD model for gabapentin. Observations vs population (A and G) or individual (D and J) predictions are represented in the left plots. The solid black line represents the identity line (y=x), the close alignment of observations with the identity line demonstrates good agreement between observed and predicted values. Scatter plot of the residuals include population weighted residuals (PWRES) versus time (B and H), individual weighted residuals (IWRES) versus time (E and K), PWRES versus population predictions (C and I), and IWRES versus individual predictions (F and L). Residuals were symmetrically distributed around zero without major trends across time or predictions, supporting the adequacy of the final model fit


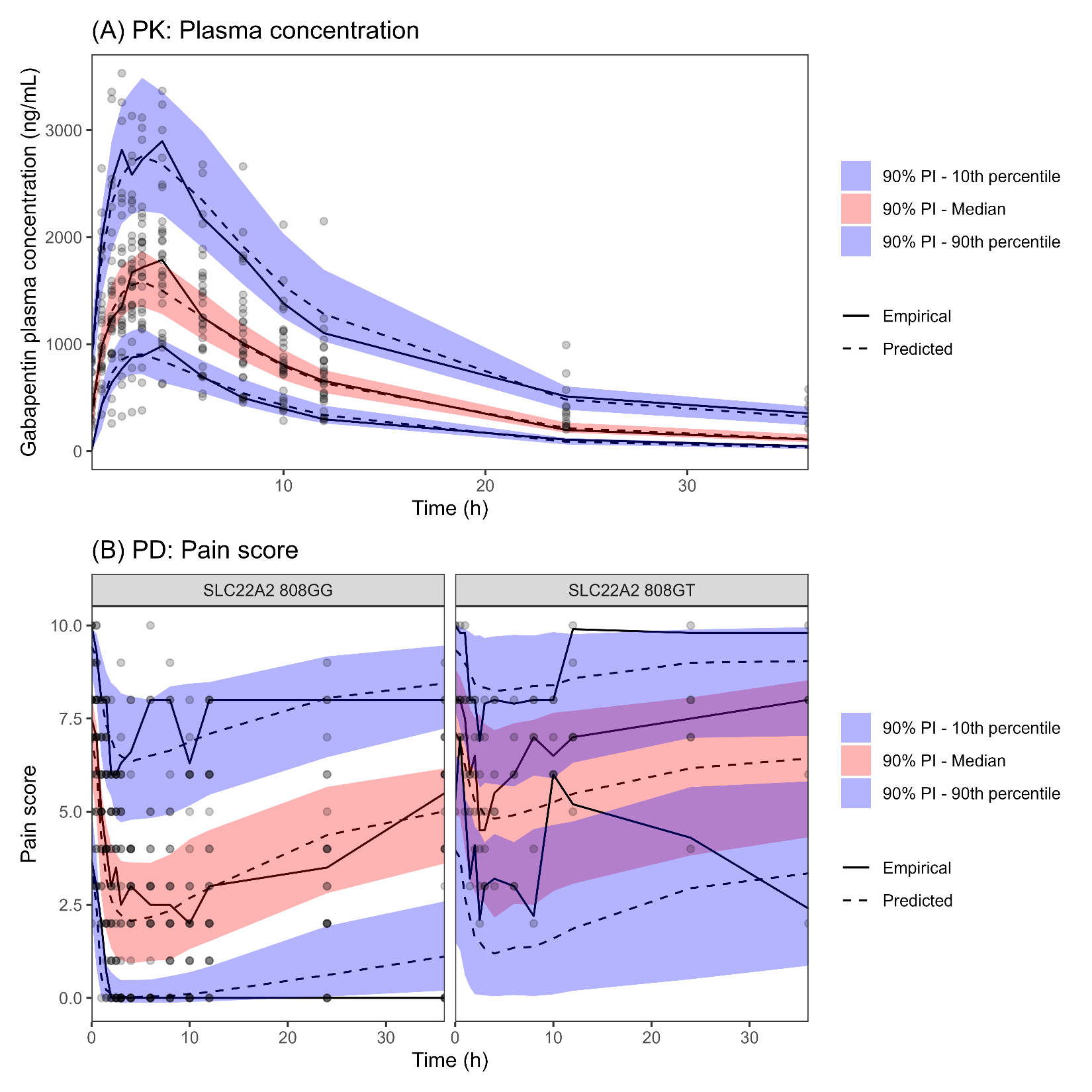


**Supplementary Figure 3.** Visual predictive check (VPC) of gabapentin plasma concentration (A) and pain score (B). Empirical and predicted percentiles are shown as solid and dashed lines, respectively. Shaded areas represent the 90% prediction intervals around the 10^th^, 50^th^, and 90^th^ predicted percentiles. Observed data are displayed as circles

**
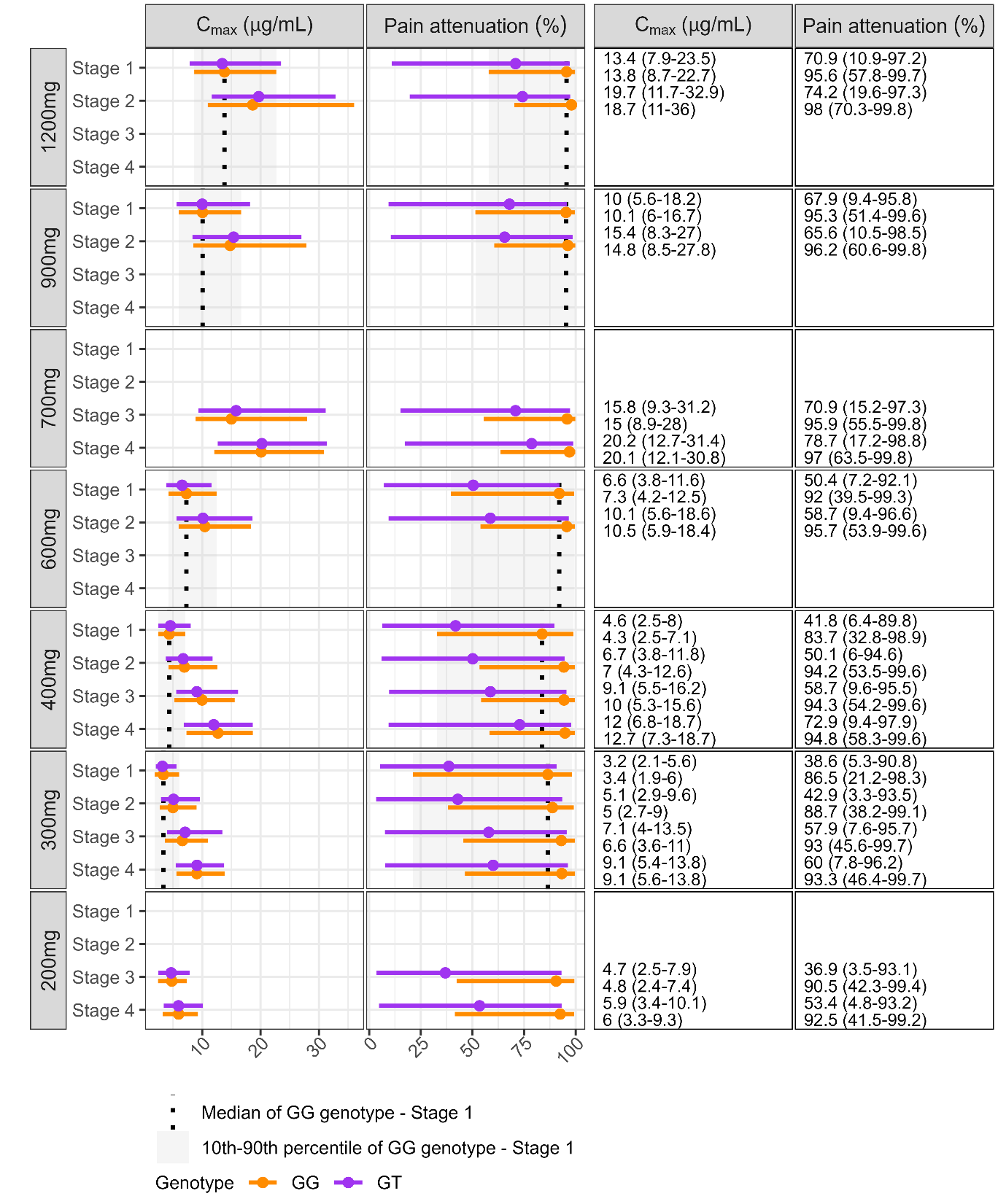
**

**Supplementary Figure 4.** Effect of *SLC22A2* c.808G>T genotype and renal function stage on the maximum concentration (C_max_) and maximum pain attenuation response at steady-state (5 weeks). Simulations of multiple dose regimen with dose ranging from 200 mg up to 1200 mg administered three times daily (TID, Stage 1 and 2), twice daily (BID, Stage 3), and once daily (QD, Stage 4). The group with *SLC22A2* GG genotype with normal or high renal function (Stage 1, eGFR > 90 mL/min/1.73 m^2^), represented as median (dotted line) and 10-90^th^ percentiles (shaded area), was used as reference group. The data is represented as median (solid circle) and 10-90^th^ percentiles (error bar), with homozygous wild-type (GG) and heterozygous (GT) genotypes in orange and purple, respectively. Renal function stages: Stage 1 – normal or high renal function (eGFR > 90 mL/min/1.73 m^2^); Stage 2 – mild decreased (60-89 mL/min/1.73 m^2^); Stage 3 – moderate to severe decreased (30-59 mL/min/1.73 m^2^); Stage 4 – severe decreased (15-29 mL/min/1.73 m^2^)

**Supplementary Data 1.**

**Final PK/PD structural model code in Monolix:**

DESCRIPTION:

PKPD model. The PK data must be tagged with the lowest OBSERVATION ID value.

The administration is extravascular with a first order absorption (rate constant ka) with a lag time (Tlag).

The PK model has a central compartment (volume V1), a peripheral compartment (volume V2, intercompartmental clearance Q), and a linear elimination (clearance Cl).

The PD model is a Imax model with effect compartment and a full inhibition (Imax=1) at high concentrations (rate constant coming in the effect compartment = ke1, coming out of the effect compartment = ke2, baseline effect E0, and half-maximal inhibitory concentration IC50).

[LONGITUDINAL]

input = {Tlag, ka, Cl, V1, Q, V2, ke1, ke2, E0, IC50, Imax}

PK:

;====== PK part of the model

; Parameter transformations

V = V1

k12 = Q/V1

k21 = Q/V2

; PK model definition and effect compartment

{Cc} = pkmodel(Tlag, ka, V, Cl, k12, k21)

EQUATION:

;====== Effect compartment with separate rate constants

; Effect compartment dynamics

ddt_Ce = ke1 * Cc - ke2 * Ce

;====== PD part of the model

E = E0 * (1 - Ce/(Ce+IC50))

OUTPUT:

output = {Cc, E, Ce}

table = {Cc, Ce, E}

**Supplementary Data 2.**

**Final PK/PD structural model code in Simulx:**

[COVARIATE]

input = {OCT2, EGFR_CKD}

OCT2 = {type=categorical, categories={'0', '1'}}

EQUATION:

logtEGFR_CKD = log(EGFR_CKD/84.8532)

[INDIVIDUAL]

input = {Cl_pop, omega_Cl, Q_pop, omega_Q, V1_pop, V2_pop, ka_pop, omega_ka, Tlag_pop, omega_Tlag, omega_V1, logtEGFR_CKD, beta_Cl_logtEGFR_CKD, E0_pop, omega_E0, IC50_pop, Imax_pop, ke1_pop, omega_ke1, ke2_pop, OCT2, beta_ke1_OCT2_1}

OCT2 = {type=categorical, categories={'0', '1'}}

DEFINITION:

Cl = {distribution=logNormal, typical=Cl_pop, covariate=logtEGFR_CKD, coefficient=beta_Cl_logtEGFR_CKD, sd=omega_Cl}

Q = {distribution=logNormal, typical=Q_pop, sd=omega_Q}

V1 = {distribution=logNormal, typical=V1_pop, sd=omega_V1}

V2 = {distribution=logNormal, typical=V2_pop, no-variability}

ka = {distribution=logNormal, typical=ka_pop, sd=omega_ka}

Tlag = {distribution=logNormal, typical=Tlag_pop, sd=omega_Tlag}

E0 = {distribution=logitNormal, max=10.1, typical=E0_pop, sd=omega_E0}

IC50 = {distribution=logNormal, typical=IC50_pop, no-variability}

Imax = {distribution=logitNormal, max=1.01, typical=Imax_pop, no-variability}

ke1 = {distribution=logNormal, typical=ke1_pop, covariate=OCT2, coefficient={0, beta_ke1_OCT2_1}, sd=omega_ke1}

ke2 = {distribution=logNormal, typical=ke2_pop, no-variability}

[LONGITUDINAL]

input = {a1, a2, b2}

;;;; Included file '2cmpt PKPD Imax with effect compt_k1ek2e.txt'

DESCRIPTION:

PKPD model. The PK data must be tagged with the lowest OBSERVATION ID value.

The administration is extravascular with a first order absorption (rate constant ka) with a lag time (Tlag).

The PK model has a central compartment (volume V1), a peripheral compartment (volume V2, intercompartmental clearance Q), and a linear elimination (clearance Cl).

The PD model is a Imax model with effect compartment and a full inhibition (Imax=1) at high concentrations (rate constant coming in the effect compartment = ke1, coming out of the effect compartment = ke2, baseline effect E0, and half-maximal inhibitory concentration IC50).

input = {Tlag, ka, Cl, V1, Q, V2, ke1, ke2, E0, IC50, Imax}

PK:

;====== PK part of the model

; Parameter transformations

V = V1

k12 = Q/V1

k21 = Q/V2

; PK model definition and effect compartment

{Cc} = pkmodel(Tlag, ka, V, Cl, k12, k21)

EQUATION:

;====== Effect compartment with separate rate constants

; Effect compartment dynamics

ddt_Ce = ke1 * Cc - ke2 * Ce

;====== PD part of the model

E = E0 * (1 - Ce/(Ce+IC50))

OUTPUT:

output = {Cc, E, Ce}

table = {Cc, Ce, E}

;;;;

DEFINITION:

y1 = {distribution=logNormal, prediction=Cc, errorModel=constant(a1)}

y2 = {distribution=logitNormal, min=-0.2, max=10.2, prediction=E, errorModel=combined1(a2, b2)}

; Additional lines

EQUATION:

RespPC = 100 - ((E/E0)*100)
